# Plasmid‐Mediated AmpC Detection in *Escherichia coli* and *Klebsiella pneumoniae*: In Vitro Prevalence and In Silico Inhibitor Design

**DOI:** 10.1155/cjid/1023166

**Published:** 2026-10-29

**Authors:** Muhammad Sarfraz, Aatif Amin, Muhammad Naveed, Mehboob Ahmed, Shahida Hasnain, Saima Naz, Mohsin Khurshid, Mohsin Tassawar Cheema, Samman Ikram, Ajaz Ahmad

**Affiliations:** ^1^ Institute of Microbiology and Molecular Genetics University of the Punjab Quaid-e-Azam Campus Lahore 54590 Pakistan pu.edu.pk; ^2^ Department of Microbiology Faculty of Science and Technology University of Central Punjab Lahore 54000 Pakistan ucp.edu.pk; ^3^ Department of Biotechnology Faculty of Science and Technology University of Central Punjab Lahore 54000 Pakistan ucp.edu.pk; ^4^ Institute of Microbiology Government College University Faisalabad Pakistan gcu.edu.pk; ^5^ Department of Cardiothoracic Surgery School of Medicine University of Pittsburgh Pittsburgh 15232 Pennsylvania USA pitt.edu; ^6^ Department of Clinical Pharmacy College of Pharmacy King Saud University Riyadh 11451 Saudi Arabia ksu.edu.sa

**Keywords:** antimicrobial resistance, *Escherichia coli*, in silico drug repurposing, *Klebsiella pneumoniae*, plasmid-mediated AmpC *β*-lactamase

## Abstract

**Objectives:**

This study investigated the prevalence of AmpC beta‐lactamase‐producing *Escherichia coli* and *Klebsiella pneumoniae* in clinical specimens collected in Lahore, Pakistan, and aimed to identify potential inhibitors targeting AmpC beta‐lactamases.

**Methods:**

A total of 17,500 clinical specimens were collected from June 2018 to June 2021 and analyzed for *E. coli* and *K. pneumoniae*. These isolates underwent phenotypic identification using microscopy and the API 20E kit. AmpC beta‐lactamase production was initially screened using cefoxitin disk diffusion and inhibitor‐based methods, while the presence of plasmid‐borne AmpC genes (CIT, EBC, and DHA) was confirmed by PCR. Additionally, the study used computational drug discovery techniques to screen for oxadiazole, quinazolinone, and sulfonylurea derivatives as potential inhibitors of the AmpC protein.

**Results:**

Our data showed high antibiotic resistance rates among *E. coli* and *K. pneumoniae* isolates: 74.5% were resistant to cefotaxime and ceftazidime, and 45% to cefoxitin. PCR testing revealed that the CIT gene was the most prevalent in both *E. coli* (65.4%) and *K. pneumoniae* (63%), while other genes (EBC and DHA) and combinations were also present (*p* < 0.005). Using computational drug discovery, several compound libraries were screened against the AmpC protein. Tolbutamide showed the most promising results, with strong binding affinity and favorable drug‐like properties.

**Conclusion:**

Plasmid‐mediated AmpC beta‐lactamase‐producing *E. coli* and *K. pneumoniae* were highly prevalent, with the CIT genotype predominating among clinical isolates. Computational screening identified Tolbutamide as a promising lead compound, warranting further experimental validation before therapeutic application.

## 1. Introduction

The *Enterobacteriaceae* family is a large group of Gram‐negative bacteria ubiquitously present in the intestines of humans and animals. These microorganisms are routinely isolated from clinical specimens and are important causes of hospital‐acquired infections in Pakistan. Among these, multidrug‐resistant *Escherichia coli* and *Klebsiella pneumoniae* are frequently identified as significant pathogens [1].

Antimicrobial resistance mediated by AmpC beta‐lactamase genes among *Enterobacteriaceae* is a significant global issue. AmpC beta‐lactamase genes are commonly found in many Gram‐negative bacteria [2] and encode cephalosporinase (AmpC), a bacterial enzyme known for its ability to hydrolyze *β*‐lactam antibiotics [3]. AmpC beta‐lactamase has been shown to demonstrate mutation‐driven adaptability to overcome even the most recent *β*‐lactam‐β‐lactamase inhibitor combinations [4]. The AmpC beta‐lactamase genes were first identified in ampicillin‐resistant *E. coli* and were later found to be located either on chromosomes or plasmids. While chromosomal AmpC *β*‐lactamases are inducible in several *Enterobacteriaceae*, *E. coli* and *K. pneumoniae* generally lack an inducible chromosomal AmpC system. Therefore, AmpC production in these species is most commonly associated with acquired plasmid‐mediated AmpC (pAmpC) genes. The presence of plasmid‐mediated AmpC genes facilitates their rapid dissemination, posing a significant public health challenge due to increasing rates of antimicrobial resistance [5]. These genes can rapidly disseminate between strains via mobile genetic elements (e.g., transposons, integrons) or through transformation and conjugation [6, 7]. The capacity of *Enterobacteriaceae* to rapidly disseminate resistance factors across different microbial species poses a substantial challenge to public health. The detection of reduced susceptibility to third‐generation cephalosporins and cefoxitin suggests AmpC production. It is noteworthy that different AmpC genes on a plasmid can result in varying resistance profiles to different antibiotics [8]. Strains carrying CMY, ACC, MOX, FOX, and DHA‐type AmpC *β*‐lactamases exhibit a range of resistance levels within *Enterobacteriaceae* [5].

The expression of mobilized plasmid‐encoded variants, such as FOX‐1 and MOX‐1, enables AmpC cephalosporinases to confer resistance to a broad spectrum of antibiotics, including most cephalosporins such as ceftazidime, ceftriaxone, cefotaxime, cefazolin, cefoxitin, and cephalothin, but excluding fourth‐generation cephalosporins and carbapenems such as meropenem/vaborbactam and imipenem/relebactam [9–11]. *E. coli* strains carrying conjugative plasmids encode DHA‐type AmpC *β*‐lactamases (DHA‐1, DHA‐33) and mediate resistance to first‐ and third‐generation cephalosporins (e.g., cephalothin, ceftazidime, cefotaxime) and cephamycins, with notable hydrolytic activity against cefotaxime [10, 12]. Plasmid‐mediated AmpC *β*‐lactamase‐producing isolates, predominantly encoded by the CMY‐2 gene (CIT group), have shown a rising trend over the past decade, especially in Asia [13]. The predominant AmpC genes identified in Southeast Asia are CMY‐2 and DHA‐1 [14]. ACC‐type AmpC enzymes are exclusively associated with Enterobacter species [15, 16].

The increasing global prevalence of plasmid‐mediated AmpC *β*‐lactamases, including those within the CIT and DHA families, highlights the critical need for effective detection methods and comprehensive molecular characterization of specific variants that impact antibiotic resistance profiles in *E. coli* and *K. pneumoniae* [17]. Key aspects such as plasmid stability, transferability, and fitness impacts under clinical and environmental pressures remain poorly understood, limiting our ability to fully elucidate the temporal evolution and geographical dissemination of antibiotic resistance across Pakistan. [18, 19].

Beyond AmpC *β*‐lactamases, other emerging resistance mechanisms also pose significant threats. One such significant challenge is the frequent colocalization of extended‐spectrum beta‐lactamases (ESBLs) and AmpC *β*‐lactamases (pAmpC) on mobile plasmids in *E. coli* and *K. pneumoniae* [20, 21]. ESBLs are sensitive to *β*‐lactamase inhibitors like clavulanic acid. Although clavulanic acid does not have antibacterial effects on its own, it can enhance the effectiveness of *β*‐lactams when used in combination with them against bacteria that produce *β*‐lactamases [22]. A critical clinical implication of this coproduction is the potential for pAmpC enzymes to mask the presence of ESBLs during routine phenotypic detection tests, leading to false‐negative results and potentially inappropriate antibiotic therapy [21, 23]. The extensive presence of ESBLs and carbapenemases on plasmids in Pakistan suggests a rich environment for the acquisition and dissemination of other plasmid‐mediated resistance genes, including pAmpC. This dual resistance mechanism complicates treatment strategies and emphasizes the need for accurate tests to ensure proper antibiotic use.

Furthermore, recent studies have also identified recurrent chromosomal mutations in siderophore‐mediated iron uptake pathways as an emerging resistance mechanism in NDM‐producing *E. coli* ST361. The NDM variant can be chromosomally integrated, but is predominantly carried on plasmids, which facilitate its horizontal dissemination [24]. During investigations targeting NDM genes, pAmpC enzymes were also incidentally detected. The widespread dissemination of NDM resistance genes has contributed to the global spread of pAmpC genes [25]. Cefiderocol—a next‐generation, last‐resort siderophore‐cephalosporin designed to evade *β*‐lactamase‐mediated hydrolysis—has shown reduced effectiveness against infections caused by these *E. coli* ST361 strains [26]. This resistance extending to crucial last‐resort agents significantly compromises the effectiveness of existing antimicrobial therapies and is proving a hurdle in developing effective strategies to combat these drug‐resistant bacteria [27–29].

Consequently, there is a pressing need for holistic genomic analyses to unravel the complex interplay of mobile genetic elements governing pAmpC gene transfer and stable maintenance in *E. coli* and *Klebsiella* populations within Pakistan [19]. Moreover, the development of novel antimicrobial agents has become a critical imperative to surmount these resistance barriers and effectively manage infections caused by resistant pathogens [30–36].

To address this critical imperative, one promising strategy to counteract bacterial *β*‐lactamase enzymes involves inhibiting their production, expression, or action to restore the activity of *β*‐lactam antibiotics. Researchers are exploring various chemical scaffolds, including derivatives of oxadiazole, quinazolinone, and sulfonylurea, for their potential to combat these challenging pathogens [37]. Oxadiazole derivatives, particularly the 1,3,4‐oxadiazole class, have demonstrated promising antibacterial activity against a range of Gram‐negative bacteria, including *E. coli* and *K. pneumoniae* [38]. Quinazolinone and its derivatives represent another important class of compounds being investigated for their antimicrobial properties, particularly against MDR Gram‐negative bacteria. Oxadiazole and quinazolinone derivatives show direct evidence of activity, and their development as chemical inhibitors is currently ongoing against multidrug‐resistant *E. coli* and *Klebsiella* [39–41]. While sulfonylurea derivatives have been explored for their antimicrobial potential, specific literature directly detailing the use or efficacy of sulfonylurea derivatives against multidrug‐resistant *E. coli* and *Klebsiella* is less prominent in recent academic literature [42–45].

The present study aimed to detect AmpC *β*‐lactamase‐producing *E. coli* and *K. pneumoniae* among clinical isolates from Lahore, Pakistan, and to investigate the genetic context of plasmid‐encoded AmpC genes to better understand their mobility and dissemination. Additionally, computational approaches were employed to identify potential drug candidates targeting AmpC *β*‐lactamases. This integrated approach may contribute to a better understanding and management of infections caused by AmpC‐producing *Enterobacteriaceae*.

## 2. Materials and Methods

The present study was carried out at the Institute of Microbiology and Molecular Genetics (MMG), University of the Punjab, Lahore, Pakistan, from June 2018 to June 2021. A total of 17,500 clinical samples were gathered from patients visiting Al Razi Laboratories in Lahore, Pakistan.

### 2.1. Ethical Considerations

Prior to commencing this research study, ethical approval was obtained from the Ethical Review Committee at Al Razi Health Care, Lahore, consisting of Dr. Sheikh Moeen‐ul‐Haq (Chief Executive Officer, Consultant Palliative Medicine), Professor Dr. Shahida Mohsin (Consultant Pathologist), and Mr. Naseem Rashid Qazi (Administrator), in accordance with the principles outlined in the Declaration of Helsinki. Furthermore, prior to the collection of clinical samples, informed consent was acquired from each participant. The informed consent process involved explaining the study details to each individual in a language they understood, after which each participant signed the consent form. Participants expressed willingness to provide samples and consented to the use of isolates for research purposes. Additionally, they were assured that their personal information would be treated confidentially and used exclusively for the stated research objectives.

### 2.2. Identification and Isolation

All the samples, including blood, urine, cerebrospinal fluid (CSF), pus, sputum, tracheal secretions, and pleural effusion, underwent processing to confirm the presence of AmpC‐positive *K. pneumoniae* and *E. coli*, as described [46]. These samples were inoculated on blood agar and MacConkey agar, while urine samples were plated on CLED agar. Phenotypic identification was carried out using the API 20E (bioMérieux) for accurate identification.

### 2.3. Antimicrobial Susceptibility Testing

Antimicrobial susceptibility testing was performed using the Kirby–Bauer disk diffusion method according to CLSI guidelines [47]. Intermediate isolates were interpreted according to CLSI recommendations and are not reported separately. The antibiotic susceptibility profile was established using the following antibiotics and their respective disk concentrations; amikacin (AK, 30 μg), cefoxitin (FOX, 30 μg), cefixime (CFM, 5 μg), ceftriaxone (CRO, 30 μg), cefotaxime (CTX, 30 μg), ceftazidime (CAZ, 30 μg), cefuroxime (CXM, 30 μg), gentamicin (CN, 10 μg), co‐amoxiclav (AMC, 20/10 μg), imipenem (IPM, 10 μg), ciprofloxacin (CIP, 5 μg), levofloxacin (LEV, 5 μg), colistin sulfate (CT, 10 μg), meropenem (MEM, 10 μg), moxifloxacin (MXF, 30 μg), piperacillin–tazobactam (TZP, 100/10 μg), and sulbactam–cefoperazone (SCF, 75/30 μg). The control strains used were ATCC 25922 for *E. coli* and ATCC 700603 for *K. pneumoniae*. Following the CLSI guidelines of 2023, isolates were categorized as resistant, intermediate, or susceptible based on the zone of inhibition. A total of 293 isolates that tested positive through the inhibitor‐based method were selected, as they were confirmed phenotypically for AmpC beta‐lactamase production [48].

### 2.4. Amplification of CIT, DHA, MOX, EBC, FOX, and ACC Genes

Genomic DNA was extracted using the boiling method. PCR was used to detect and amplify six AmpC genes, including CIT, DHA, MOX, EBC, FOX, and ACC. The PCR kit used was Thermo Scientific DreamTaq Green PCR Master Mix (2×). The DreamTaq Green PCR Master Mix (2×) was thawed and instantly vortexed for a minute. The PCR tube was kept chilled, and all the components were added to make the total mixture volume 50 μL, and the mixture was vortexed and spun down. The PCR conditions observed were according to DreamTaq Green PCR Master Mix (2×) recommendations. A single cycle of initial denaturation was run at 95°C for 1–3 min, with 35 amplification cycles consisting of denaturation at 95°C for 30 s, annealing at 5°C less than melting temperature (*T*_*m*_), and extension at 72°C for 1 min, and final extension for 5–15 min at 72°C. The primer sequence and Tm are provided in Table 1. Bacterial strains were screened for the presence of all six resistance genes (CIT, DHA, MOX, EBC, FOX, and ACC), and results were focused on those positively detected. The analysis of PCR‐amplified genes was performed using agarose gel electrophoresis with a 1.5% agarose gel in TAE buffer, to which SYBR Green staining dye was added. A 6× loading dye (1 μL) was mixed with 5 μL of PCR product. A 1500‐bp DNA ladder was run in the first well as a size marker, and the gel was run at 100 V till the dye reached three‐quarters of the gel’s length (∼40 min). The gel was observed under the G‐Box UV transilluminator.

**TABLE 1:** Primers used for the PCR amplification of AmpC genes.

Primers AmpC		Sequence	Product size (bp)	Melting point (°C)	Nucleotide positions	GenBank Accession #
CIT	CIT‐F1	TGG CCA GAA CTG ACA GGC AAA	462	87.5	337–357	X91840
	CIT‐R1	TTT CTC CTG AAC GTG GCT GGC			778–798	

MOX	MOX‐F	GCT GCT CAA GGA GCA CAG GAT	520	90.7	126–146	X92508
	MOX‐R1	CAC ATT GAC ATA GGT GTG GTG C			625–646	

FOX	FOX‐F	AAC ATG GGG TAT CAG GGA GAT G	190	88.7	775–796	X77455
	FOX‐R	CAA AGC GCG TAA CCG GAT TGG			944–964	

DHA	DHA‐F	AAC TTT CAC AGG TGT GCT GGG T	218	87.6	258–279	Y16410
	DHA‐R	GCT GCC ACT GCT GAT AGA A			457–476	

ACC	ACC‐F	GTG CAA GCC AAT ATG GGG CAG	120	82.6	769–789	A3133121
	ACC‐R	CTC CCA CAT CAG ATC CTG AGT			868–888	

EBC	EBC‐F1	TCG GTA AAG CCG ATG TTG CGG	302	88.9	188–208	U58495
	EBC‐R1	CTT CCA CTG CGG CTG CCA GTT			469–489	

### 2.5. Phylogenetic Analysis

The phylogenetic relationships of the AmpC genes of *K. pneumoniae* and *E. coli* to the corresponding related genes were determined with BLASTn in NCBI. MEGA‐X software, Ver.05 (https://www.megasoftware.net/), was used for the construction of a phylogenetic tree by the neighbor‐joining method.

### 2.6. In Silico Analysis

Following molecular identification of the predominant plasmid‐mediated AmpC genes, computational screening was undertaken to identify potential inhibitors targeting the corresponding AmpC enzyme. This integrated approach linked epidemiological surveillance with rational inhibitor discovery.

### 2.7. Retrieval of the 3D Structure of the Protein

The 3D structure of the AmpC protein was retrieved from the Protein Data Bank (PDB), which is available at https://www.rcsb.org/. The 3D structure of a protein, DNA, RNA, or small molecule complex can be found in the freely accessible PDB [49].

### 2.8. Validation of 3D Structure

The 3D structure of the AmpC protein was validated by the online server PROCHECK which is available at https://saves.mbi.ucla.edu/. The stereochemical properties and residue geometry of a protein are assessed using PROCHECK[50]. The positions of the preferred regions were analyzed based on the Ramachandran plot regions to assess Ramachandran plot [51].

### 2.9. Protein Interaction Networks

STRING (search tool for the retrieval of interacting genes/proteins) was used to find out how the *AmpC* protein interacts with other proteins, which are available at https://string-db.org/. STRING is a freely available, regularly updated resource for the general audience [52].

### 2.10. Selection of Compounds

In this study, a diverse set of ligands and control compounds were carefully selected to assess the potential of Tolbutamide and other derivatives as inhibitors of AmpC *β*‐lactamase. The ligands chosen for docking with AmpC beta‐lactamase included tolbutamide, benzoyleneurea, 3′‐methylacetanilide, citrate, and sorbitol. Tolbutamide, a sulfonylurea primarily used to treat diabetes, was included to explore its repurposing potential as an AmpC inhibitor due to its chemical characteristics that could potentially interact with the enzyme’s active site. Benzoyleneurea and 3′‐methylacetanilide were selected for their unique chemical structures and potential inhibitory effects against AmpC *β*‐lactamase, offering new possibilities for targeting this critical resistance mechanism. Citrate and sorbitol, both naturally occurring compounds, were included to investigate their functional groups and potential to bind to the enzyme, providing a more holistic exploration of diverse chemical classes. Alongside these experimental ligands, we included sulbactam, tazobactam, and clavulanic acid as control compounds to provide a standard for comparison. These three compounds are well‐established beta‐lactamase inhibitors that have been extensively studied for their effectiveness against AmpC‐producing bacteria. Sulbactam is widely used in combination with beta‐lactam antibiotics to extend their efficacy, while tazobactam and clavulanic acid are similarly employed in clinical settings to inhibit beta‐lactamase enzymes, making them ideal benchmarks in this study. By comparing the binding affinities and docking results of the experimental ligands with these control compounds, we can evaluate the potential of tolbutamide and other compounds as inhibitors of AmpC beta‐lactamase and assess whether they show comparable or superior efficacy to these established treatments. This comprehensive approach allows us to not only explore novel inhibitors but also validate the repurposing potential of tolbutamide and similar compounds in the fight against antimicrobial resistance. All compounds were retrieved from PubChem, which is available at https://pubchem.ncbi.nlm.nih.gov [53].

### 2.11. Screening of Compounds

Five different inhibitory compounds were screened through PyRx, which is a versatile tool for screening compounds. This offline tool is used in computational drug research and discovery as a virtual screening tool to check drug target libraries for potential responses [54]. The compound with the highest energy was selected for further analysis.

### 2.12. Interaction Analysis

The protein–ligand interaction profiler (PLIP) was used to determine the bond length of the docked complex and is available at https://plip-tool.biotec.tu-dresden.de/plip-web/plip/index. It is the most cited online server used for the interpretation of bond length. [55]. It predicted interactions between the receptor and ligand, including hydrogen bonds and van der Waals interactions [56].

### 2.13. ADMET Analysis

In silico ADMET and drug‐likeness analyses were performed using SwissADME. It is an online platform for conducting preliminary clinical trials of potential new medications and is available at http://www.swissadme.ch/. It predicted key characteristics like toxicity, dispersion, metabolism, and absorption [57]. Drug‐like properties of the molecule were determined using its optimized PDB structure as input [58].

### 2.14. Statistical Analysis

Data were analyzed using IBM SPSS Statistics Version 26.0 (IBM Corp., Armonk, NY, USA). Descriptive statistics were used to summarize the prevalence of bacterial isolates, antimicrobial resistance patterns, and AmpC gene distribution. Results are presented as frequencies and percentages. Comparisons between *E. coli* and *K. pneumoniae* were performed using Pearson’s chi‐square test. A two‐tailed *p* value of < 0.05 was considered statistically significant.

## 3. Results

Of the 17,500 clinical specimens including blood, urine, CSF, pus, sputum, tracheal secretions, and pleural effusion, 29% yielded positive bacterial cultures. Among the bacterial isolates recovered, *E. coli* accounted for 61% and *K. pneumoniae* for 39%. High rates of resistance to antibiotics (cefotaxime or ceftazidime) were found: 58% in *E. coli* and 42% in *K. pneumoniae*. Many resistant strains also showed resistance to cefoxitin, with 53% of *E. coli* and 46% of *K. pneumoniae*. Phenotypic testing confirmed AmpC beta‐lactamase production in 25.6% of *E. coli* and 74% of *K. pneumoniae* isolates, and the prevalence of pAmpC‐positive isolates did not differ significantly between *E. coli* and *K. pneumoniae* (*χ* ^2^ = 0.645, *p* = 0.422), as shown in Table 2. PCR (molecular testing) results for AmpC genes: 27% *E. coli* and 73% *K. pneumoniae* among positive results. The most common AmpC gene detected was CIT (63.7%), followed by EBC (14.7%), and combinations like CIT + EBC (10.5%) and CIT + DHA (6.3%). No MOX, FOX, or ACC genes were found (Figure S1a & S1b). The distribution of genes among *E. coli* and *K. pneumoniae* is summarized in Table 3 and is shown in the graphical picture in Figure 1a. Although the CIT genotype was the predominant AmpC gene family in both bacterial species, the overall distribution of AmpC gene families did not differ significantly between *E. coli* and *K. pneumoniae* (*χ* ^2^ = 7.681, *p* = 0.104).

**TABLE 2:** Prevalence of pAmpC in *E. coli* and *K. pneumoniae*.

	Number	Percentage
*E. coli (n = 75)*		
pAmpC positive	52	69%
Non‐pAmpC	23	31%

*K. pneumoniae* (*n* = 218)		
pAmpC positive	138	63%
Non‐pAmpC	80	37%

**TABLE 3:** Distribution of CIT, DHA, and EBC among *E. coli* and *K. pneumoniae* isolates.

Bacterial isolate	CIT	DHA	EBC	CIT + DHA	CIT + EBC
*E. coli*	34	0	11	1	6
*K. pneumoniae*	87	9	17	11	14

**FIGURE 1:**
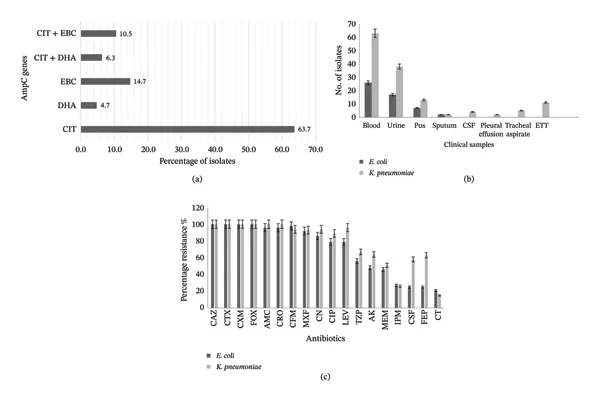
(a) Distribution of genes among *E. coli* and *K. pneumoniae*, (b) isolation of AmpC‐positive isolates from different specimens, and (c) resistance pattern of selected isolates against different antibiotics.

AmpC‐positive isolates were more frequent in male patients (56%) than females (44%) and were mainly recovered from blood (47%) and urine (29%) (Figure 1b). All AmpC‐positive *E. coli* and *K. pneumoniae* were resistant to several key antibiotics, including ceftazidime, cefotaxime, cefuroxime, and cefoxitin. *E. coli* AmpC producers had high resistance to cefixime (98%), co‐amoxiclav (96%), ceftriaxone (96%), moxifloxacin (92%), gentamicin (86%), ciprofloxacin (79%), and levofloxacin (79%), with lower resistance to other antibiotics. *K. pneumoniae* AmpC producers were universally resistant to co‐amoxiclav and ceftriaxone (100%), and showed high resistance to levofloxacin (96%), cefixime (94%), gentamicin (94%), moxifloxacin (93%), and ciprofloxacin (89%), with moderate to lower resistance to other drugs (Figure 1c).

### 3.1. Phylogenetic Analysis

In the phylogenetic analysis, the selected strain of *K. pneumoniae* (X91840) showed 100% homology with *K. pneumoniae* strain WCHKP7E2 (CP028804), and this clade showed 99% similarity with *K. pneumoniae* strain (K191773). Likewise, the phylogenetic relationship of the EBC genotype of *K. pneumoniae* (U58495) had 99% homology with *K. pneumoniae* (MCQ‐95). This clade had 98% similarity with *K. pneumoniae* (U58495). Similarly, the selected *E. coli* strain showed 98% and 100% homology with the (AmpC) CMY and EBC genes, respectively. The trees were constructed using the neighbor‐joining method, and bootstrap values were calculated from 1000 tree replicates (Figure 2a,b).

**FIGURE 2:**
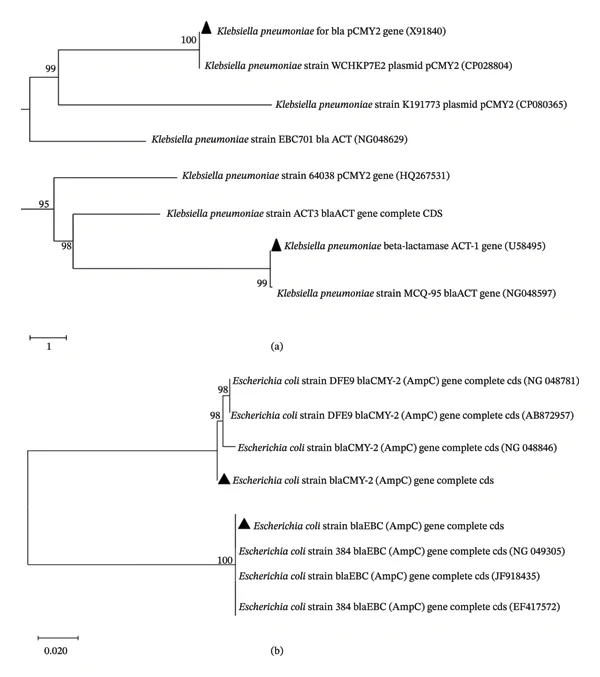
Phylogenetic tree of selected bacterial strains based on 16S rRNA gene sequencing, constructed to interpret the evolutionary history of the organisms by neighbor‐joining method. (a) *K. pneumoniae* and (b) *E. coli*.

### 3.2. Retrieval of the 3D Structure of Protein

The 3D structure of the AmpC protein corresponding to the predominant CIT gene family was retrieved from the PDB under PDB ID 1C3B. The structure comprises 358 amino acids and was used for subsequent structural validation and in silico inhibitor analysis, as shown in Figure 3a.

**FIGURE 3:**
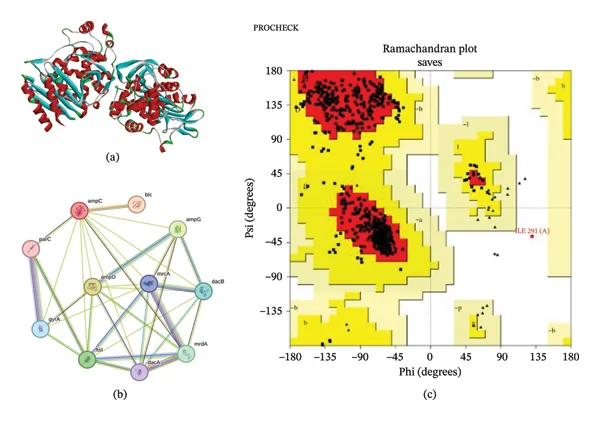
Computational analysis of the *AmpC* protein. (a) Retrieval of 3D structure, (b) validation of the Ramachandran plot, and (c) protein interaction pathway.

### 3.3. Validation of 3D Structure

The 3D structure of the AmpC protein was predicted through PROCHECK. The predicted model showed an ERRAT value of 94.84, indicating the stability of the protein. The Ramachandran plot showed that 543 residues were present in most favored regions, that is, 90.2%; 58 residues were present in additional allowed regions, that is, 9.6%; and only 1 residue was present in disallowed regions, that is, 0.2%. The Ramachandran plot is shown in Figure 3b.

### 3.4. Protein Interaction Networks

Visualization of the interaction of the *AmpC* protein with other proteins was accomplished utilizing STRING (search engine for the retrieval of interacting genes/proteins). The *AmpC* protein had 11 nodes with direct links to other proteins like *blc, ampD, gyrA, parC, dacA,* and *mrdA.* The *AmpC* protein had higher interaction with *blc, ampD, and ampG*, with scores of about 0.96, 0.91, and 0.90. The pathway of the *AmpC* protein is shown in Figure 3c.

### 3.5. Selection of Compounds

All inhibitory compounds were retrieved from PubChem, and their PDB structures were used for further analysis. The PDB structures of these selected compounds and known inhibitors are shown in Supporting Table 2.

### 3.6. Screening of Compounds

To filter out the best candidates, five inhibitory compounds and three already known inhibitors were docked against the *AmpC* protein using PyRx. The binding affinities of the selected compounds for AmpC beta‐lactamase revealed varying degrees of inhibition. Tolbutamide showed the strongest binding affinity at −8.0 kcal/mol, suggesting its potential as an AmpC inhibitor. Benzoyleneurea (−7.2 kcal/mol) and 3′‐methylacetanilide (−6.2 kcal/mol) also displayed moderate binding, while citrate (−6.0 kcal/mol) and sorbitol (−5.2 kcal/mol) showed weaker affinities. Among the known inhibitors, Sulbactam (−6.3 kcal/mol) and tazobactam (−7.0 kcal/mol) had strong affinities, while clavulanic acid exhibited the weakest binding at −6.1 kcal/mol. These results suggest that tolbutamide emerged as a promising computational lead compound. The list of binding affinities of screened compounds is shown in Supporting Table 1.

Based on binding energies, the compound with the most favorable (lowest) binding energy, such as tolbutamide, was selected. Using AutoDock Vina, the docking was performed between the *AmpC* protein and Tolbutamide. The docked complex is shown in Figure 4a.

**FIGURE 4:**
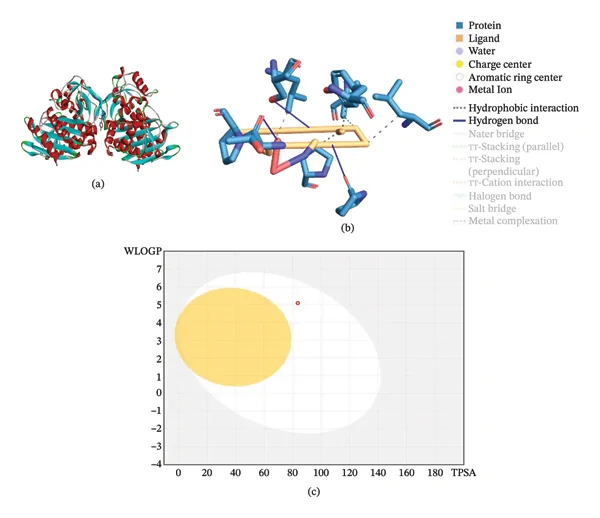
Screening and interaction analysis of AmpC protein. (a) Docked complex of *AmpC* protein and tolbutamide, (b) protein–ligand interaction profiler, and (c) boiled egg model to investigate the absorption of small compounds through the gastrointestinal tract.

### 3.7. Interaction Analysis

PLIP provided further interpretation of the interaction data by estimating bond length. As the typical length of a hydrogen bond is between 2.7 and 3.3 angstroms, the three hydrogen bonds detected were 2.87, 2.7, and 2.99 angstroms. Due to the slightly greater distances associated with van der Waals interactions (3.3–4.0 angstroms), it was projected that three van der Waals or hydrophobic bonds were present, with lengths of 3.57, 3.60, and 3.77 angstroms, respectively. The interactions predicted by PLIP are shown in Figure 4b.

### 3.8. ADMET Analysis

The physicochemical properties of the selected compound, as represented by SwissADME, included its gastrointestinal (GI) absorption, water solubility, bioavailability score, and synthetic accessibility, which are depicted in Supporting Table 3. There were no violations of Lipinski’s rule of five, as indicated by the international standard drug‐likeness rule. The ADMET parameters of selected tolbutamide are shown in Supporting Table 3.

### 3.9. Boiled Egg Model

The SwissADME server predicted the boiled egg model. The boiled egg model provides a rapid, intuitive, easily repeatable, remarkably robust technique to investigate the absorption of small compounds through the GI tract and the entry of those compounds into the brain; this is very useful for the development and administration of medications. It is speculated that molecules can penetrate the blood–brain barrier if they are located in the yellow section of the boiled egg model, while those located in the white area of the egg will be absorbed by the digestive system. If a molecule is located in the white region, it can pass through the digestive system, as shown in Figure 4c.

## 4. Discussion

The escalating antibiotic resistance among Gram‐negative bacteria poses a substantial threat to human health. *E. coli*, within the *Enterobacteriaceae* family, emerges as a critical pathogen, frequently detected in various samples and notably responsible for urinary tract infections [59, 60]. In our results, *E. coli* (61%) and *K. pneumoniae* (39%) stand out as the predominant bacterial species recovered, responsible for life‐threatening infections in both male and female patients of all ages. Likewise, the prevalence rate of *E. coli* among the highly reported uropathogens from cities in Punjab ranges from 35% to 73%, followed by *K. pneumoniae* at 18%–27% [61–64].

Our work focused on isolates of *E. coli* and *K. pneumoniae* from 17,500 clinical specimens, including blood, urine, pus, sputum, CSF, ETT, pleural effusion, and tracheal aspirate from Al Razi Health Care laboratories and their outreach collection centers throughout Lahore. Both *E. coli* and *K. pneumoniae* strains in our work were predominantly recovered from blood specimens, constituting the highest percentages at 46.7% and 45.9% (*p* < 0.005), respectively. In this work, a higher ratio of resistant isolates was recovered from male patients compared to females, data that mirror the finding from another study carried out at Ittefaq Hospital Lahore [65].

The global occurrence of multidrug‐resistant bacterial pathogens is a concerning issue, particularly in developing countries like Pakistan, where resources are limited, and antibiotic misuse exacerbates resistance. Recent reports from tertiary care hospitals in Pakistan estimated that 69%–92% of bacteria resistant to third‐generation cephalosporins are found in patients with different types of infections [63, 64]. A number of studies conducted in Pakistan have focused on extended‐spectrum *β*‐lactamases, carbapenemases, monobactams, and broad‐spectrum cephalosporins to understand the action of beta‐lactamase enzymes. The rising resistance in MDR strains across the world is attributed to the proliferation of ESBLs [66], and Pakistani studies confirm the widespread genetic prevalence of ESBLs in Gram‐negative rods from clinical specimens [67] and their presence in *K. pneumoniae* across human, animal, and environmental sources [68].

To identify ESBL production in the target *K. pneumoniae* and *E. coli* strains, the double‐disk synergy test (DDST) was performed following CLSI guidelines. Out of 293 bacterial isolates, 52.2% were confirmed as ESBL producers. Specifically, 61.3% of *E. coli* strains and 49.1% of *K. pneumoniae* strains were ESBL‐positive. Since our study is primarily focused on the genetic analysis of AmpC genes, we did not conduct a detailed investigation of the genetic profile of ESBL‐producing strains. Our phenotypic analysis results are consistent with other studies reporting a high frequency of ESBL‐producing *E. coli* and *K. pneumoniae* from Pakistan. [64] detected the presence of 76.1% *E. coli* and 64.3% *K. pneumoniae* among the ESBL‐producing pathogens isolated from UTI patients in a tertiary care hospital in Pakistan using the DDST. Molecular characterization of ESBL producers by researchers has reported the presence of CTX genes to be most frequent, with CTX‐M (56.5%), followed by TEM (28.3%), and SHV (10.9%); coexistence of CTX‐M/TEM was observed in two isolates from four Tehsils of District Jhang, Pakistan [69]. In Ayub Medical Hospital, Abbottabad, Pakistan, a team of researchers found 28.57% of ESBL‐producing strains to be carrying CTX‐M‐15%, and 14.29% carried TEM‐1 genes among others (SHV‐1, SHV‐75, and OXA‐1). Research from Peshawar indicated a high prevalence of ESBL‐producing *E. coli*, with prominent genotypes including CTX‐M, TEM, and SHV. These isolates demonstrated resistance to cephalosporins, quinolones, and sulfonamides [70]. Similarly, a study from Lahore documented the emergence of MDR *K. pneumoniae* strains coharboring carbapenemase genes (e.g., KPC‐2, NDM‐1, OXA‐48) alongside ESBL genes (CTX‐M, SHV). Similar findings about the molecular profile of ESBL producers across the globe have been reported elsewhere. In Iraq, the CTX‐M gene was reported to be the most common genotype present universally in 76%–100% of the ESBL‐producing species, followed by TEM, detected molecularly in 52%–75% of the isolates [71]. In Ethiopia, the ESBL gene CTX‐M‐15 in *E. coli* isolates of human origin is strongly associated with the pandemic *E. coli* clone O25:H4‐ST131 [72].

A high prevalence of resistance to third‐generation cephalosporins has been consistently reported in *E. coli* and *K. pneumoniae* isolates from Pakistan [73]. This phenotypic resistance, while often attributed to ESBLs, can also be conferred by AmpC *β*‐lactamases. AmpC beta‐lactamase is an enzyme produced primarily by Gram‐negative bacteria, particularly *Enterobacteriaceae*, which can hinder the effectiveness of antibiotics and is insensitive to common *β*‐lactamase inhibitors such as clavulanic acid used for ESBL detection. The significant association of resistance genes with plasmid replicons draws attention to the role of mobile genetic elements in their dissemination [74]. The data concerning antimicrobial resistance related to AmpC‐producing *K. pneumoniae* and *E. coli* in clinical settings from Pakistan remain limited. To fill this research gap, the present study investigates the occurrence and screening of plasmid‐mediated AmpC *β*‐lactamase‐producing *E. coli* and *K. pneumoniae* isolates from patients in Lahore, Pakistan.

According to our results, 69% of *E. coli* and 63% of *K. pneumoniae* tested positive for AmpC beta‐lactamase. Among 293 AmpC *β*‐lactamase‐positive isolates, 244 (83%) were identified as pAmpC‐positive by the modified cefoxitin Hodge test (MHT), while 190 (65%) were subsequently confirmed as pAmpC beta‐lactamase producers through PCR. For *K. pneumoniae*, PCR confirmed 53 of 70 (76%) MHT‐positive isolates. Similarly, for *E. coli*, 48 of 69 (70%) phenotypically positive strains were PCR‐confirmed as pAmpC producers. In contrast to our findings, lower prevalence rates of pAmpC *β*‐lactamases have been reported in India, with 5% in *K. pneumoniae* and 18% in *E. coli* [25]. Likewise, the pooled prevalence of AmpC‐producing *E. coli* and *K. pneumoniae* in Africa was reported to be 15.3% and 17.3%, respectively [75]. The dissemination of ESBL‐ and pAmpC‐carrying strains of *E. coli* and *K. pneumoniae*, which harbor the CIT (CMY‐2, likely originating from the chromosomal cephalosporinase of *Citrobacter freundii*), EBC, DHA, MOX, FOX, and ACC pAmpC genes, stands as the primary driver of the antibiotic resistance that has emerged in recent years [76]. According to the molecular analyses of detected pAmpC variants in this research work, the CIT gene was found to be the most prevalent among isolates, followed by EBC. Comparable results were observed in the Indian study, and CMY‐42 and DHA‐1 were found to be the dominant pAmpC variants, with persistent detection of CMY‐42 [25]. The CIT + EBC combination was the third most common, followed by CIT + DHA. The DHA gene alone was the least frequent. However, no MOX, FOX, or ACC genes were detected in any of the phenotypically confirmed pAmpC producer strains.

Furthermore, these AmpC enzymes are being frequently detected along with other critical resistance mechanisms, such as ESBLs and carbapenemases.

Because AmpC‐producing bacteria may exhibit reduced susceptibility to carbapenems, particularly when additional resistance mechanisms are present, there is an urgent need for new antibiotics and alternative treatments. One such alternative is the use of mechanism‐based inhibitory compounds, which can mimic the structure of *β*‐lactams and can significantly reduce the production and efficacy of the *β*‐lactamase‐based drug resistance. Three such inhibitor molecules available commercially are clavulanic acid, sulbactam, and tazobactam [77]. However, their inhibitor activity is limited mostly to class A, and there remains a need to identify novel inhibitory compounds against class C *β*‐lactamase enzymes. Similarly, researchers have successfully used oxadiazoles–sulfonamide complexes to identify motifs with enhanced antimicrobial potential against both Gram‐negative and Gram‐positive bacteria [78]. The antimicrobial activity and toxicity of these chemical derivatives are largely dependent on the structure and the position of the functional group or their respective substituent in the benzene ring. Tolbutamide, a first‐generation sulfonylurea derivative, was selected. Tolbutamide is a first‐generation sulfonylurea primarily used as a hypoglycemic agent. In the present study, it was investigated as a potential AmpC inhibitor based solely on computational predictions. These novel compounds have demonstrated promising results in docking studies, ADME (absorption, distribution, metabolism, and excretion), toxicity evaluations, and other in silico analyses, offering valuable insights for drug design to address the escalating problem of drug resistance. Therefore, tolbutamide should currently be considered only as a computationally predicted lead compound requiring comprehensive microbiological, biochemical, and in vivo validation before any therapeutic application.

### 4.1. Limitations of the Study

This study has several limitations that should be considered when interpreting the findings. First, there are currently no internationally standardized recommendations for the routine screening and molecular confirmation of plasmid‐mediated AmpC beta‐lactamase genes in clinical microbiology laboratories. This lack of harmonized guidelines makes the consistent detection and surveillance of plasmid‐mediated AmpC‐producing isolates challenging across healthcare settings. Furthermore, this was a single‐region study based on convenience sampling, and although the results provide valuable insights into the prevalence and molecular characteristics of AmpC‐producing *E. coli* and *K. pneumoniae* in Lahore, Pakistan, they may not fully represent the epidemiology of other regions. Nationwide or multicenter surveillance studies are, therefore, warranted to better understand the geographic distribution and dissemination of these resistance determinants.

In addition, molecular characterization was limited to PCR‐based detection of selected AmpC gene families. Whole‐genome sequencing or plasmid sequencing was beyond the scope of the present study but would provide more comprehensive information regarding plasmid diversity, gene variants, mobile genetic elements, and transmission dynamics.

The computational component of this study included molecular docking, protein validation, protein–ligand interaction analysis, and ADMET prediction to identify potential AmpC inhibitors. However, molecular dynamics simulations and binding free‐energy calculations were not performed, and these approaches may provide additional insights into the stability and strength of protein–ligand interactions. Furthermore, although Tolbutamide demonstrated favorable binding affinity toward AmpC beta‐lactamase in silico, it is primarily an antidiabetic sulfonylurea with no established antimicrobial activity. Therefore, it should be regarded as a computational lead compound rather than a confirmed therapeutic candidate. Comprehensive in vitro enzyme inhibition assays, antimicrobial susceptibility studies, in vivo investigations, and clinical validation are required before its potential as an AmpC beta‐lactamase inhibitor can be confirmed.

## 5. Conclusion

This study provides comprehensive epidemiological, molecular, and computational insights into plasmid‐mediated AmpC beta‐lactamase‐producing *E. coli* and *K. pneumoniae* isolated from clinical specimens in Lahore, Pakistan. The high prevalence of the CIT genotype, together with extensive resistance to *β*‐lactam antibiotics, highlights the growing challenge posed by AmpC‐mediated antimicrobial resistance and emphasizes the need for continuous molecular surveillance and effective antimicrobial stewardship. The integration of molecular detection with computational drug screening identified Tolbutamide as a promising computational lead compound exhibiting favorable binding affinity and drug‐like properties against AmpC beta‐lactamase. However, these findings are based solely on in silico analyses and should be interpreted cautiously. Comprehensive in vitro, in vivo, and clinical investigations are required to validate its inhibitory activity and therapeutic potential. Overall, this study enhances our understanding of plasmid‐mediated AmpC dissemination and provides a foundation for future studies aimed at developing novel strategies to combat beta‐lactamase‐mediated antimicrobial resistance.

**Nomenclature**
AmpC: Ambler class C inducible cephalosporinase
MDR: Multidrug resistance
ESBL: Extended‐spectrum *β*‐lactamase
ADMET: Absorption, distribution, metabolism, excretion, and toxicity
NCBI: National Center for Biotechnology Information
CLSI: Clinical and Laboratory Standards Institute

## Author Contributions

Muhammad Sarfraz executed the experimental work. Aatif Amin wrote, proofread, and submitted the draft. Muhammad Naveed performed in silico analyses. Mehboob Ahmed analyzed the experimental data, and Shahida Hasnain supervised the research project. Saima Naz and Samman Ikram revised the draft. Ajaz Ahmad provided the funding for open‐access publication. Mohsin Tassawar Cheema and Mohsin Khurshid reviewed the final draft for submission.

## Funding

This work was supported by the Ongoing Research Funding Program (ORF‐2026‐350), King Saud University, Riyadh, Saudi Arabia.

## Disclosure

All authors have given consent for publication of this manuscript.

## Conflicts of Interest

The authors declare no conflicts of interest.

## Supporting Information

Additional supporting information can be found online in the Supporting Information section.

## Supporting information


**Supporting Information** Figure S1. PCR amplification of CIT, EBC, and DHA genes in *K. pneumoniae* and *E. coli*. Supporting Table 1. List of binding affinities of compounds with targeted AmpC protein. Supporting Table 2. PDB structures of selected inhibitory compounds. Supporting Table 3. ADMET parameters of tolbutamide.

## Data Availability

The data that support the findings of this study are available in the supporting information of this article.
