# Supplementary material for: Plasmid‐Mediated AmpC Detection in Escherichia coli and Klebsiella pneumoniae: In Vitro Prevalence and In Silico Inhibitor Design
Source: Can J Infect Dis Med Microbiol. 2026 Oct 29;2026:1023166. doi: 10.1155/cjid/1023166 (PMC13717588; doi:10.1155/cjid/1023166)
Supplement: Supplementary file 1 [file CJID-2026-1023166-s001.docx]

**Supplementary Materials**

**Plasmid-Mediated AmpC Detection in *Escherichia coli* and *Klebsiella pneumoniae*: *In Vitro* Prevalence and *In Silico* Inhibitor Design**

Muhammad Sarfraz^1^, Aatif Amin^2*^, Muhammad Naveed^3^, Mehboob Ahmed^1^ and Shahida Hasnain^1^, Saima Naz^2^, Mohsin Khurshid^4^_,_ Mohsin Tassawar Cheema^5^_,_ Samman Ikram^2^ and Ajaz Ahmad^6^

^1^Institute of Microbiology and Molecular Genetics, University of the Punjab, Quaid-e-Azam Campus, Lahore 54590, Pakistan

^2^Department of Microbiology, Faculty of Science and Technology, University of Central Punjab, Lahore 54000, Pakistan

^3^Department of Biotechnology, Faculty of Science and Technology, University of Central Punjab, Lahore 54000, Pakistan

^4^Institute of Microbiology, Government College University Faisalabad, Pakistan

^5^Department of Cardiothoracic Surgery, School of Medicine, University of Pittsburgh, Pittsburgh, PA 15232, USA

^6^Department of Clinical Pharmacy, College of Pharmacy, King Saud University, 11451 Riyadh, Saudi Arabia

**Running title:** Plasmid-Encoded AmpC Detection and β-Lactamase Inhibitor Design

*Author for correspondence: Dr. Aatif Amin

Email: [aatif.amin@ucp.edu.pk](mailto:aatif.amin@ucp.edu.pk)

**Figure S1.** PCR amplification of *cit, ebc* and *dha* genes in *K. pneumoniae* and *E. coli.*

**Supplementary Table 1** List of binding affinities of compounds with targeted AmpC protein

| Sr. No | Compounds | Compounds Binding affinity (Kcal/mol) |
| --- | --- | --- |
| 1 | Tolbutamide | -8.0 |
| 2 | Benzoyleneurea | -7.2 |
| 3 | 3'-Methylacetanilide | -6.2 |
| 4 | Citrate | -6.0 |
| 5 | Sorbitol | -5.2 |
| 6 | Sulbactam | -6.3 |
| 7 | Tazobactam | -7.0 |
| 8 | Clavulanic acid | -6.1 |

**Supplementary Table 2** PDB structures of selected inhibitory compounds and controls

| **Sr. No.** | **Compounds** | **Structures** |
| --- | --- | --- |
| **1.** | Tolbutamide |  |
| **2.** | Sorbitol |  |
| **3.** | Citrate |  |
| **4.** | Benzoyleneurea |  |
| **5.** | 3'-Methylacetanilide |  |
| **6.** | Sulbactam |  |
| **7.** | Tazobactam |  |
| **8.** | Clavulanic acid |  |

**Supplementary Table 3** ADMET parameters of Tolbutamide

| Formula | C13H18O2 |
| --- | --- |
| Molecular weight | 294.54 g/mol |
| Num. heavy atoms | 18 |
| Num. arom. heavy atoms | 0 |
| Fraction Csp3 | 0.92 |
| Num. rotatable bonds | 7 |
| Num. H-bond acceptors | 3 |
| Num. H-bond donors | 2 |
| Molar Refractivity | 72.84 |
| TPSA (Topological Polar Surface Area) | 83.65 Å² |
| Water Solubility Log S (ESOL) | -2.62 |
| GI absorption | High |
| Skin Permeation (Log K p ) | -6.50 cm/s |
| Bioavailability Score | 0.55 |
| Synthetic Accessibility | 4.71 |
| Lipinski’s rule of 5 | No violation |
